# Effects of Response Force Parameters on Medial-Frontal Negativity

**DOI:** 10.1371/journal.pone.0054681

**Published:** 2013-01-22

**Authors:** Anne-Simone Armbrecht, Henning Gibbons, Jutta Stahl

**Affiliations:** 1 Department of Psychology, University of Cologne, Cologne, Germany; 2 Institute for Psychology, University of Bonn, Bonn, Germany; University of Gent, Belgium

## Abstract

The response-related medial-frontal activity (MFN) is often supposed to reflect action-monitoring and error-processing activity. The present force-production task was designed to investigate the effects of two response parameters (i.e., peak response force and time-to-peak, TTP) on the MFN separately. In a 2 × 2 design (high vs. low target force and short vs. long TTP), 22 participants had to produce isometric force pulses to match one of four conditions (e.g., a high target force with a long TTP). Significant main effects of both target force and target TTP were revealed. As previously shown, the MFN amplitude was higher in the high target-force condition than in the low target-force condition. Contrary to the initial expectations, a long TTP had the effect of reducing the MFN amplitude. There was no error-specific effect on the MFN. The force-unit monitoring model (FUMM) is suggested to account for the force- and TTP- specific variations of MFN amplitude, latency and slope.

## Introduction

Action monitoring is an important cognitive function. It controls the coordination and the adjustment of behavioral processes to prevent negative outcomes like response errors. Many studies have investigated how the cognitive system monitors the selection and execution of (erroneous) distinct actions like left-hand and right-hand responses (e.g., [1]), but only a few studies have focused on the effects of (erroneous) *continuous* movement parameters like the magnitude of response force on the monitoring activity [2]–[5]. In everyday life, the production of the appropriate degree of force is an important and sometimes challenging task. The motor system often has to adapt instantaneously. For example, the consequences would be enormous if a neurosurgeon uses the scalpel with an incorrect degree of force while cutting into a patient’s prefrontal cortex, or if a crane operator produces an incorrect force on the joystick while loading a container of toxic waste. Therefore, a fast and well-functioning force-sensitive monitoring system is inevitably required. However, error detection seems to be much more demanding in continuous tasks compared to distinct motor tasks. There is some evidence in the literature that the medial-frontal cortex is involved in force monitoring [2]–[5].

The medial-frontal cortex, in particular the anterior cingulate cortex (ACC), is assumed to play an essential role in action monitoring (e.g., [6], [7]), and has been revealed as the neural generator of the error(-related) negativity (Ne/ERN) [8]. The Ne/ERN is a negative component in the event-related potential (ERP), peaking between 50 and 100 ms after response errors [1], [4], and it has been suggested that it reflects an internal error detection signal [1] or response-conflict monitoring [9]. Several studies also reported an Ne/ERN-like component after correct responses (correct-response negativity; CRN), for example, in ambiguous response situations (e.g., a flanker task [10]). As the two ERP components seem to be generated in the rostral areas of the ACC and are thus assumed to reflect the same (or at least similar) processing mechanisms [11], we use the label *MFN* to refer to these two components as *medial-frontal negativity* in the following (see also [12]).

In a four-choice force-production task, de Bruijn et al. [3] investigated the effects of different force magnitudes and force production errors on the monitoring activity systematically. The participants had to decide between a left-hand and a right-hand response and between a low or high target force (low target force: 14% to 26% of maximum voluntary force, MVF; high target force: 32% to 56% of MVF). The MVF for each index finger was assessed at the beginning of the experiment to define specific force criteria separately for each participant. In contrast to previous studies [4], [5], de Bruijn et al. [3] reported a higher MFN amplitude after responses in the high target-force condition than in the low target-force condition, and suggested that the monitoring system is sensitive to the force magnitude. The authors further differentiated between different force-error types: only errors of *force selection* (i.e., the exerted response force was opposite to the target force; e.g., in the low target-force condition, the response force was too high) but not errors of *force execution* (i.e., the exerted response force was incorrect, but not opposite to the target force; e.g., in the low target-force condition, the response force was too low) led to a higher MFN amplitude. The authors assumed that only errors of force selection could be detected by the monitoring system, because the exerted response force differed strongly from the target force, and hence involved an incorrect choice between discrete behavioral processes (i.e., responding with a low or a high response force). As the exerted response force of the errors of force execution differed only slightly from the required target force, the monitoring system was not able to detect the deviation and the MFN amplitude did not increase.

In our previous study [2], we tried to support a force-error sensitive monitoring system in a unimanual force production task (to prevent the additional appearance of hand errors) by using similar target-force conditions (low target force: 15% to 25% of MVF; high target force: 34% to 56% of MVF). However, we did not find a higher MFN amplitude after forceful responses than after weak responses, and there was no difference between the MFN amplitudes after force errors and correct responses, indicating that the monitoring system might be insensitive to the effects of force magnitude and force errors.

### Objective of the Present Study

Due to the contradictory results of the above-mentioned studies [2], [3], we wanted to investigate the effects of force productions on the monitoring system more specifically. Although the two studies used similar individual target-force ranges (see above), which had to be reached by the peak of the force pulses, the temporal dynamics of the force productions, as reflected by the time between the response onset and the peak of the force pulse (*time to peak*, *TTP*), were not controlled. The observed TTP variations within and between the two studies ([2]: low target force: 136±9 ms; high target force: 174±11 ms; [3]: low target force: 154±45 ms; high target force: 192±62 ms) indicate that different force production mechanisms might have been involved in the different tasks as postulated in the *parallel force unit model* (PFUM) [13].

According to the PFUM, a force pulse, and therefore a specific response force, is produced by the sequenced activation of force units. A force unit implies the activation of a motor neuron, innervating specific muscle fibers. The authors of the PFUM [13] suggested different mechanisms for reaching a specific response force. The activated force units can vary in either number (the more force units are activated, the higher the response force) or duration of activation (the longer the force units are activated, the higher the response force) to exert a specific response force (for details see [13], [14]). Even if the same maximum response force is exerted with either of these two mechanisms, the corresponding force-time curves will not show the same dynamics. A prolonged activation of the set of force units affects the slope of the force-time curve negatively (i.e., it becomes less steep) and the TTP increases. This means that if two force pulses show similar response forces but different TTPs (as mentioned above, [2]–[3]), two different mechanisms might have been involved in the force productions.

Applying the assumptions of the PFUM to our previous study [2], the behavioral findings (a rather short mean TTP and small standard deviation) indicated that the force production mainly resulted from variations in the number of activated force units compared to de Bruijn et al.’s study [3]. The high standard deviations of TTP in their study indicate that different types of force production mechanisms were involved in the two target-force conditions. Thus, high response forces might have been produced by prolonging the duration of activation of the force units. As the authors reported a higher MFN amplitude after high response force (including correct responses in the high target-force condition and errors of force selection in the low target-force condition) than after low response forces, the monitoring system might be sensitive to the different force production mechanisms. This would indicate a relationship between the monitoring system and temporal adjustments of force productions indicated by longer TTPs. One could assume that the later the target force was reached, the longer monitoring was necessary. Findings of longer MFN latencies after responses with prolonged TTPs (i.e., errors of force selection compared to correct responses [3], and responses with a high response force compared to the low response force [2]) also supported this assumption. These considerations raised the question of whether the ACC monitors the temporal adjustments of the force-production mechanisms instead of monitoring the correct or incorrect *selection* of force magnitude.

In the present study, we aimed to investigate the relationship between the MFN and force-production mechanisms by systematically varying the maximum of the response force and the TTP. If the monitoring system is sensitive to temporal adjustments of force-production mechanisms, it should increase its activity after responses with a long TTP compared to a short TTP, as long TTPs imply a prolonged activation of the force units (see PFUM [13]). If the monitoring system is sensitive to the force magnitude, responses with a high response force would result in a higher MFN than responses with a low response force, irrespective of the force production mechanism (i.e., after short and long TTPs). Finally, it might be a combination of both mechanisms.

Although force-specific error processing is not the main research question of the present study, we wanted to compare our data with the results of former studies [2]–[5] and thus, investigated errors of force production additionally. In order to increase the accuracy of force production in combination with long and short TTP, we employed a blocked design (i.e., one target force/target TTP within a block, for details see Method). Due to this modification the error types in our study are different from the errors in these studies. We cannot differ between error of selection and error of execution but between an *error opposite to the target force (target TTP)* and an *error not opposite to the target force (target TTP).*

## Methods

### Participants

Twenty-two participants (12 females), all students from the Georg August University of Goettingen, were tested in the present study. The participants were paid (€ 7.50 per hour) or received course credits for their participation. Their ages ranged from 19 to 33 years (mean ± SD: 23±0.8 years). All participants were right-handed and reported normal or corrected-to-normal vision. Informed written consent was given. This study was approved by the ethics committee of the German Psychological Association (DGPs).

### Procedure and Experimental Task

In order to provide the formation of an internal representation of the target-force and target-TTP ranges and therefore a high amount of correct force pulses, the participants practiced the experimental task (without EEG application) the day before the EEG experiment. For the sake of brevity, we only describe the procedure of the EEG experiment. The two experimental sessions differed only in the number of practice and experimental trials.

#### Practicing force pulses

The session started with two practice trials for each index finger to accustom the participants to producing brisk, isometric force pulses in each trial with force sensitive response keys (see below; sampling frequency: 250 Hz). The waveforms of the force pulses were simultaneously presented on the monitor to provide simultaneous visual feedback on the performance. On average, the participants were able to produce three force pulses within each practice trial (i.e., 5000 ms). The next trial was initiated by a key press with the resting hand. Each force-time curve was further controlled for deviations (i.e., multiple or long-lasting peaks, an incomplete resolution of the force pulse), which were shown to the participants, and it was explained that such responses had to be excluded from further analyses.

#### Maximum voluntary force

In order to calculate individual target forces, the MVF was determined for each index finger separately by pressing the force key as hard as possible without moving the forearm. The key press was indicated for 300 ms by three parallel crosses (visual angle of 0.5°), which were surrounded by a continuously present white frame (horizontal angle of 2.4° and vertical angle of 1.9°) in the center of a black screen. The MVF was calculated by averaging seven trials for each hand. A low target force was defined as 20% of the MVF and a high target force as 45% of the MVF. We defined two target-force ranges, for which the widths were set according to Weber’s law (*k* = ΔR/R), to account for the different task difficulties between the high and low response forces. Higher response forces lead to higher standard deviations which means that it is more difficult to hit the required target force (e.g., [15], [16]; for a simulation study see [17]). The range widths (*ΔR*) were calculated by setting *R* as the calculated target force (20% or 45% of the MVF) of each hand and *k* = 0.5 (see [18]–[19]; low target-force range: 15–25% of the MVF; high target-force range: 34–56% of the MVF). The short target-TTP range was set from 113 to 188 ms and the long target-TTP range from 300 to 500 ms, as our preliminary experiments had shown acceptable performance rates for these values and ranges [18].

#### Practicing the target forces and target TTPs

**Figure 1:**
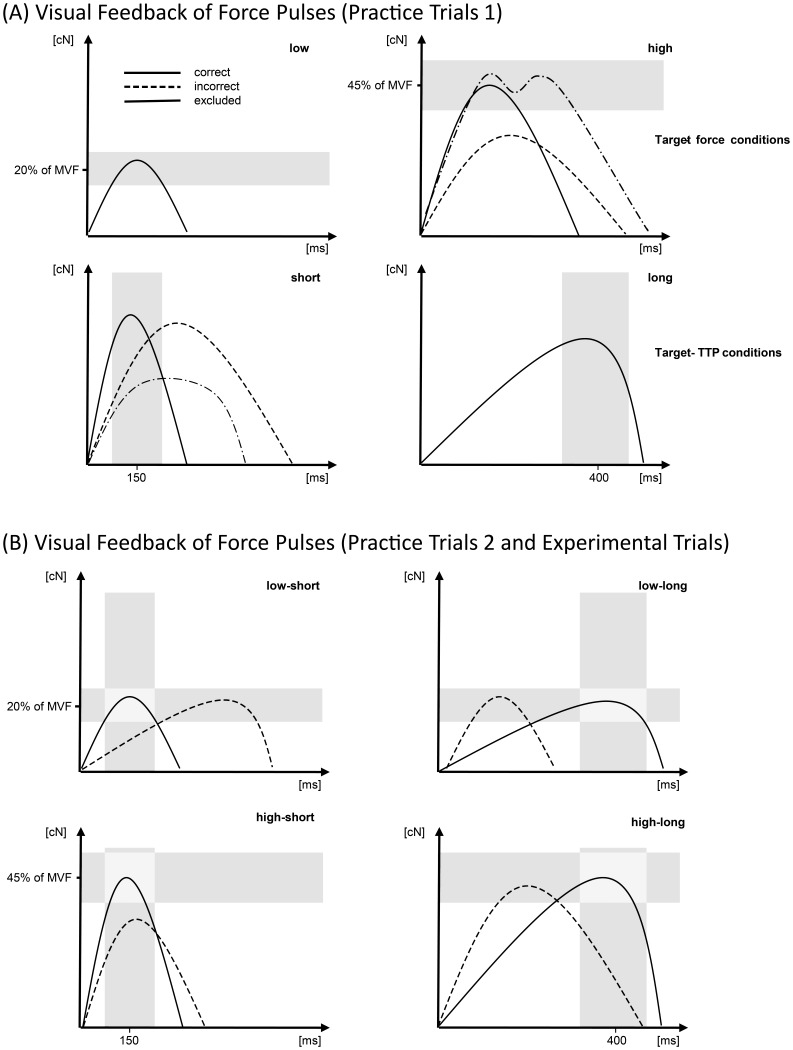
Visual feedback of the force-time diagrams. (A) Force-time diagrams of the first practice trials including the target-force ranges (shaded areas) for 20% and 45% of MVF (upper panels) and target-TTP ranges for 150 ms and 400 ms (lower panels). A correct and an incorrect response, as well as an insufficient, excluded force pulse, are depicted. (B) Force-time diagrams for the four experimental conditions obtained by the combinations of the two target-force and the two target-TTP ranges.

In order to provide a high level of task performance, several practice trials were conducted before the experiment began. The range of each target force was presented as a horizontal bar and the range of each TTP as a vertical bar in the force-time diagram for visual feedback (see Figure 1A). The range of the TTPs was presented with response onset, as they could not be calculated before response onset. The participants were again instructed to produce brisk, isometric force pulses that should peak within the presented target-force range or within the presented TTP range (examples of correct, incorrect, and excluded force pulses are given in Figure 1A). The target forces and target TTPs were practiced in two trials for each target force, TTP, and hand.

The combinations of each target force with each target TTP comprised the four experimental conditions (i.e., low–short, low–long, high–short, high–long), which were practiced in two further practice trials for each hand (i.e., the force pulse had to peak within the rectangle of the target-force and target-TTP ranges; see Figure 1B).

#### Experimental trials

The experimental trials had to be performed by each participant without the simultaneous presentation of the force-time curve in order to prevent the effects of monitoring of the visual information. The experiment comprised 16 blocks (30 trials per block), that is, four blocks (two for each hand) for each condition. Within one block only one of the four conditions was realized. The first five trials of each block served as practice trials and were excluded from further analyses. Hence, 100 trials per condition were presented, resulting in 400 experimental trials.

A row of three white crosses (+++) on a black background was presented for 300 ms as an imperative stimulus in the center of the white frame. Each of the four conditions was performed in two consecutive blocks with the left and the right hand. The order of the conditions (i.e., of the blocks) was fully balanced between the participants. Visual feedback was presented 2000 ms after the stimulus onset, indicating whether the exerted force pulse was within the corresponding target ranges. A correct force production was indicated by the appearance of a green frame for 600 ms, and an incorrect force production by a red frame.

In order to provide precise feedback, knowledge of results (KR) was given for 1200 ms after feedback offset by the presentation of the exerted force pulse in the force-time diagram including the target rectangle colored in red or green (see Figure 1B). Thus, KR indicated, for example, whether the exerted force pulse peaked too late (i.e., the force-time curve peaked on the right side of the target field, cf. Figure 1B) or was too weak (i.e., the force-time curve peaked under the target rectangle). The offset of the KR was initiated by a key press and the next trial started after 1200 ms. The instructions emphasized response speed and accuracy equally.

### Data Acquisition

#### Behavioral data

Behavioral data were recorded by means of force-sensitive keys. Each key was composed of a plastic cuboid (110 × 19 × 2 mm) attached to a spring steel plate held by an adjustable metal clamp at one end. During the experiment, the fingertip of the index finger rested on the cuboid at the open end. The response force of the index finger was measured by strain gauges at the fixed end. The analogous signal was digitized at a sampling rate of 500 Hz for an interval of 3100 ms starting with the frame presentation. Each apparatus was fixed to a board to adjust the keys to the length of the forearms and palms, which rested on the boards. The boards were located on the left and right sides of the computer screen. In order to maintain the participant’s posture during force production, an adjustable chin rest was provided (fixed at 56 cm from the screen) at a convenient height.

Reaction time (*RT*) was defined as the temporal interval from stimulus onset to the first point in time exceeding a response force of 50 cN. The parameters of the force productions were measured by the maximum of the response force (peak force, *PF*), which is defined as the peak amplitude of the force-time curve in each trial, and by the temporal delay between the response onset and the time of reaching the PF (TTP).

As the dependent behavioral measures, the percentage of correct responses (PCR), mean RT, mean TTP, and mean PF were determined separately for each participant.

#### Electrophysiological data

The EEG was recorded from 27 scalp electrode sites (FP1, FP2, F7, F3, Fz, F4, F8, FT7, FC3, FCz, FC4, FT8, T3, C3, Cz, C4, T4, CP3, CPz, CP4, T5, P3, Pz, P4, T6, O1, O2) according to the standard international 10–20 system [20]. The Ag/AgCl electrodes were referenced against the right mastoid. Vertical and horizontal electrooculograms (EOG) were recorded from electrode positions supra- and infra-orbital to the right eye and 2 cm lateral from the outer canthi. Electrodes were re-referenced off-line against algebraically linked mastoids. The EEG was continuously recorded at a sampling rate of 500 Hz using a NeuroScan Inc. data acquisition unit. A band-pass filter (0.10 Hz –70 Hz) was employed for all channels.

The electrophysiological data were averaged by using (a) the response onset and (b) the TTP as references. The EEG was analyzed off-line with epochs ranging from 400 ms before until 600 ms after response onset. A baseline period of 100 ms preceding the response onset was used before the averaging (for both response- and TTP-locked averaging). All data were screened for artifacts, and contaminated trials exceeding maximum/minimum amplitudes of ±150 µV were rejected. Influences of eye movements were eliminated by applying an ocular correction algorithm [21]. Average waveforms were further smoothed by low-pass filtering below 20 Hz.

A current source density (CSD) analysis of the ERP waveforms was performed. The CSD analysis accounted for the curvature of the head using a spline algorithm [22]–[24]. The signal is independent of the location of the reference and the effect of neighboring currents is reduced. The CSD signals were computed for each electrode site by taking the second derivative of the distribution of the voltage over the scalp.

As dependent electrophysiological measures, amplitudes and latencies of the MFN were determined separately from individual mean CSD-ERP waveforms for the Target Force (low, high), Target TTP (short, long), and Response Type (correct, incorrect). The MFN was determined at FCz, where it was most prominent (see Results). The response-locked MFN amplitude was defined as the mean, rectified amplitude for the CSD-ERP waveforms within a time window from 0 to 200 ms after response onset. The latencies of the response-locked MFN were defined separately as the period of time between the response onset and the peak amplitude (determined in the range of 0 to 150 ms after response onset). In addition, we investigate the component’s rise and decay by slope estimates. They were yielded in each condition by fitting a regression line from baseline to peak (rise) and from peak to baseline (decay). For the TTP-locked analyses, we used a peak-to-peak analysis as the intervals to determine the peak amplitude as the peak latency of the component strongly differ between the conditions.

### Statistical Analyses

All statistical analyses involved analyses of variance (ANOVAs) with repeated measures for behavioral and electrophysiological data. A two-way ANOVA was conducted for mean PCR, including the within-subject factors *Target Force* and *Target TTP*. Three-way ANOVAs were calculated separately for mean RT, mean TTP, mean PF, MFN latencies, mean amplitudes of the MFN, and slopes of MFN including the within-subject factors *Target Force, Target TTP,* and *Response Type*. The levels of significance were adjusted to correct for possible violations of sphericity [25]. All reported post-hoc tests were Tukey’s HSD tests. The level of significance was set as *p*<.05.

## Results

### Behavioral Data

**Figure 2:**
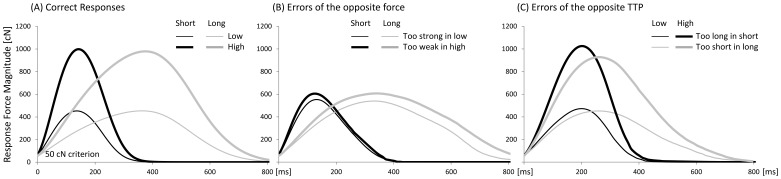
Mean force-time curves. Time-locked to response onset as a function of target force and target TTP for (A) correct responses, (B) errors of the opposite force production and (C) errors of the opposite TTP production.

Figure 2A depicts the mean force-time curves of the correct responses separately for each target force and target TTP. Figure 2 B–C shows the mean force-time curves of the different errors types (for details of the analyses, see below). The mean response rates (PCR and error rates) and standard errors of means (SEM) of the correct and different incorrect responses for each condition are presented in Table 1. In 2.6% of all trials either no response was given or the response was not a proper isometric force pulse. A two-way ANOVA performed on the PCR revealed a significant main effect of Target Force, *F*(1, 21) = 28.0, *p*<.001. The PCR was higher for responses in the high target-force condition (69.0±2.2%) than in the low target-force condition (58.7±2.9%; *p*<.001). Target TTP had no main effect on PCR (*p*>.1). A significant interaction between Target Force and Target TTP, *F*(1, 21) = 4.8, *p*<.05, showed that the difference in PCR between the low and high target-force conditions was higher in the short (13.3%) than in the long (7.3%) TTP condition (Table 1, first column; *p*<.01). We did not perform a separate analysis for the different incorrect responses as they are not independent.

**Table 1:** Mean response rates and standard errors of means (± SEM) of correct and incorrect responses as a function of Target Force (low, high) and Target TTP (short, long).

Target TTP	Correct [%]		Pure Force Error[%]		Pure TTP Error [%]	TTP-Force Error [%]
*Low Target Force*						
** Short**	**56.8±2.8**	***Total***	**16.6±1.6**	***Total***	**17.5±1.5**	**7.5±1.1**
		*Too week*	3.5±0.6	*Too short*	9.1±1.9	0.2–3.0^1^
		*Too strong*	13.0±1.7	*Too long*	8.4±1.7	
** Long**	**60.6±3.5**	***Total***	**11.9±1.3**	***Total***	**19.1±2.0**	**4.9±1.0**
		*Too week*	1.7±0.5	*Too short*	11.8±2.3	0.6–2.6^1^
		*Too strong*	10.2±1.4	*Too long*	7.3±1.4	
*High Target Force*						
** Short**	**70.1±2.0**	***Total***	**13.9±1.2**	***Total***	**11.9±1.2**	**2.8±0.5**
		*Too week*	6.4±0.8	*Too short*	2.8±0.9	0.1–1.9^1^
		*Too strong*	7.5±1.1	*Too long*	9.1±1.4	
** Long**	**67.9±3.0**	***Total***	**7.9±1.0**	***Total***	**16.6±1.8**	**3.0±0.5**
		*Too week*	4.6±1.1	*Too short*	6.9±1.5	0.5–1.2^1^
		*Too strong*	3.3±0.8	*Too long*	9.7±1.7	

Note: The values do not add up to 100% because of the excluded responses (i.e., no response and no isometric force pulses);

1 Ranges of the percentage of the four combined TTP-Force error types (e.g., too weak and too short).

The mean RTs, TTPs, PFs, and SEMs of the correct and incorrect responses (averaged across all error types) of all conditions are presented in Table 2. The three-way ANOVA revealed an effect of Target Force on RT, *F*(1, 21) = 25.3, *p*<.001. The responses were faster in the high target-force condition (295±22 ms) than in the low target-force condition (318±23 ms). No further significant main effects or interactions were obtained.

**Table 2:** Mean reaction times (RTs), time-to-peaks (TTPs), peak response forces (PFs), and standard errors of means (± SEM) for correct and incorrect responses as a function of Target Force (low, high) and Target TTP (short, long).

Target TTP	Response Type	RT [ms]	TTP [ms]	PF [cN]
*Low Target Force*				
** Short**	**Correct**	314±25.8	144±2.3	466±14.7
	**Incorrect** 1	317±23.5	153±6.3	507±17.7
** Long**	**Correct**	319±22.7	384±4.7	468±14.8
	**Incorrect** 1	324±24.2	381±14.2	515±20.1
*High Target Force*				
** Short**	**Correct**	287±22.5	150±2.3	1027±33.4
	**Incorrect^2^**	283±20.9	167±5.4	1076±42.6
** long**	**Correct**	305±23.9	396±5.3	1015±34.8
	**Incorrect^2^**	305±24.6	426±15.2	1019±54.5

1 including errors of the opposite force and errors of the not-opposite force;

^2^including errors of the opposite and errors of the not-opposite TTP.

The three-way ANOVA for TTP revealed a significant main effect of Target TTP, *F*(1, 21) = 694.0, *p*<.001. As expected, mean TTP was longer in the long target-TTP condition (397±8.8 ms) than in the short target-TTP condition (153±3.7 ms). In addition, there were significant main effects of Target Force, *F*(1, 21) = 19.7, *p*<.001 and Response Type, *F*(1, 21) = 6.0, *p*<.05, revealing longer mean TTP in the high target-force condition (285±5.7 ms) compared to the low target-force condition (266±5.0 ms), as well as longer mean TTP for incorrect responses (282±7.5 ms) than for correct responses (269±2.6 ms). A significant interaction of Target Force and Response Type, *F*(1, 21) = 10.5, *p*<.01, revealed that the longest TTPs occurred for incorrect responses in the high target-force condition (296±8.8 ms) compared to all remaining conditions (high target force: correct responses: 273±2.9 ms; low target force: correct responses: 264±2.7 ms, incorrect responses: 267±7.8 ms; all *p*s <.001). A further significant interaction of Target Force and Target TTP, *F*(1, 21) = 6.4, *p*<.05, showed that mean TTP in the long target-TTP condition was longer for strong responses (411±10.1 ms) than for weak responses (383±9.0 ms; *p*<.001). The three-way interaction between Target Force, Target TTP, and Response Type, *F*(1, 21) = 5.1, *p*<.05, further revealed the largest difference in TTP between correct and incorrect responses for the high target-force, long target-TTP condition (Table 2, second column, *p*<.01). No further significant interaction was obtained.

The three-way ANOVA conducted on PF showed a significant main effect of Target Force, *F*(1, 21) = 466.6, *p*<.001. The responses were stronger in the high target-force condition (1034±39.3 cN) than in the low target-force condition (489±15.9 cN). A main effect of Response Type, *F*(1, 21) = 9.1, *p*<.01, showed that the incorrect responses (779±31.2 cN) were stronger than the correct responses (744±23.8 cN). A three-way interaction between Target Force, Target TTP, and Response Type, *F*(1, 21) = 4.6, *p*<.05, revealed that the error-specific increment of PF was shown for all target-force and target-TTP conditions (all *p*s <.01) except for the high target-force, long target-TTP condition (Table 2, third column, *p*>.1). No further significant main effects or interactions were found.

### Electrophysiological Data

**Figure 3:**
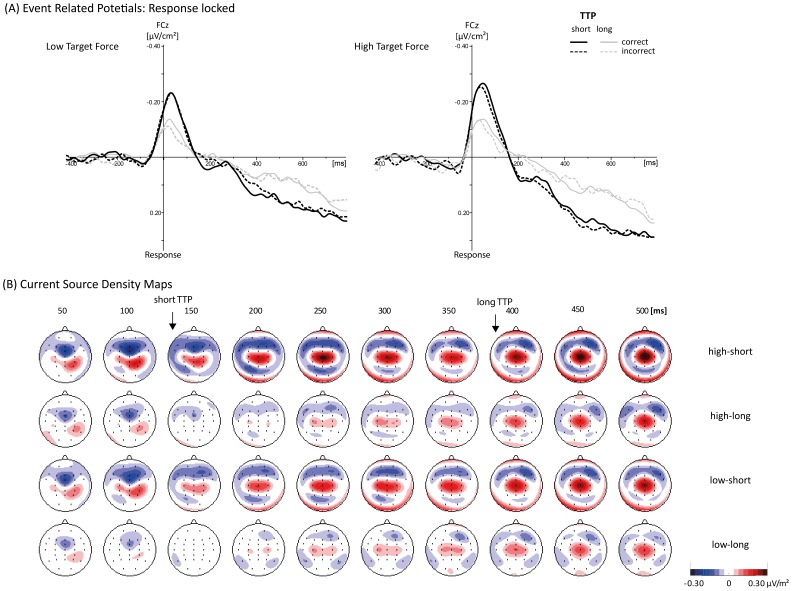
Grand average ERP waveforms and current density maps. (A) Grand average waveforms of event-related potentials at the FCz electrode site, time-locked to response onset, as a function of target TTP and Response Type (including all error types). Waveforms are shown separately for the low force condition (left panel) and high force condition (right panel). (B) Current-source density maps show the general scalp distribution of cortical activity of the correct responses separately for target forces (low, high) and target TTPs (short, long) in an interval from 50 ms to 500 ms after response onset.

Figure 3A shows the CSD-ERP waveforms as a function of Target Force and Target TTP for correct and incorrect responses at the FCz electrode site, as the current-source density map (Figure 3B) show a clear fronto-central distribution for all conditions. The Target Force had a significant effect on the mean (rectified) MFN amplitude, *F*(1, 21) = 16.4, *p*<.001. The MFN amplitude was higher after responses in the high target-force condition (0.128±0.012 µV/cm^2^) compared to the low target-force condition (0.101±0.008 µV/cm^2^). A further significant main effect of Target TTP was also found, *F*(1, 21) = 39.5, *p*<.001. This revealed that the MFN amplitude was smaller after responses in the long target-TTP condition (0.141±0.013 µV/cm^2^) compared to the short target-TTP condition (0.089±0.008 µV/cm^2^). No further significant main effects or interactions were found. The same effects were obtained by analyzing the usual ERP waveforms and also using a peak detection method (0 to 150 ms after response onset). For the sake of brevity these results are not reported.

The three-way ANOVA performed on the MFN latencies yielded a significant main effect of Target Force, *F*(1, 21) = 4.6, *p*<.05. The MFN peaked later after responses in the high target-force condition (57±5.0 ms) compared to the low target-force condition (51±4.5 ms). A significant interaction was shown for Response Type and Target Force, *F*(1, 21) = 4.6, *p*<.05. The correct responses in the high target-force condition (60±5.1 ms) were significantly higher than in all the other conditions (correct low target force: 51±5.1 ms; incorrect low target force: 51±5.4 ms; incorrect high target force: 52±5.4 ms). No further significant main effects or interactions were obtained.

Visual inspection showed variations in the rise and the decay of the MFN components across conditions (Figure 3A). Therefore, we also performed two further three-way ANOVAs for the slopes of the ascending parts and the slopes of the descending parts. The Target Force affected the rise of the MFN amplitude, *F*(1, 21) = 7.4, *p*<.05. The ascending slopes were larger in the high target-force condition (0.0058±0.0003 µV/[cm^2^·ms]) than in the low target-force condition (0.0042±0.0002 µV/[cm^2^·ms]). The Target TTP had also a significant effect on the rise of the MFN amplitude, *F*(1, 21) = 25.4, *p*<.001, with a larger ascending slope for short (0.0074±0.0006 µV/[cm^2^·ms]) than for long target TTP (0.0027±0.0002 µV/[cm^2^·ms]). No further significant effects were found.

For the descending slopes of the decay of the MFN amplitudes, a significant main effect of Target Force was revealed, *F*(1, 21) = 8.9, *p*<.01. A less negative descending slope was found for the low target-force condition (−0.0029±0.0002 µV/[cm^2^·ms]) compared to the high target-force condition (−0.0042±0.0002 µV/[cm^2^·ms]). The Target TTP had a highly significant effect on the descending slope, *F*(1, 21) = 25.7, *p*<.001, with a more negative slope for the short TTP (−0.00457±0.0003 µV/[cm^2^·ms]) than for the long TTP (−0.00252±0.0002 µV/[cm^2^·ms]). No further significant main effects or interactions were found.

### Error Types

#### Response-locked average

As mentioned earlier, we could not differentiate between errors of force selection and errors of force execution in the present task, because no selection process was required in the blocked design. Thus, we tried to differentiate between *errors opposite to the target force/target TTP* (e.g., high target force, but too weak response) and *errors not opposite to the target force/target TTP* (e.g., high target force but too strong response). As in some of the twelve error conditions only five participants showed an acceptable number of *errors not opposite to the target*, we decided to contrast only *errors opposite to the target* with correct responses. Although there was no force selection in the present task, *errors opposite to the target* were obtained similarly compared to the MFN-increasing error types of de Bruijn et al.’s study [3]. In the following, we denoted these error types as errors of the opposite force and errors of the opposite TTP.

**Figure 4:**
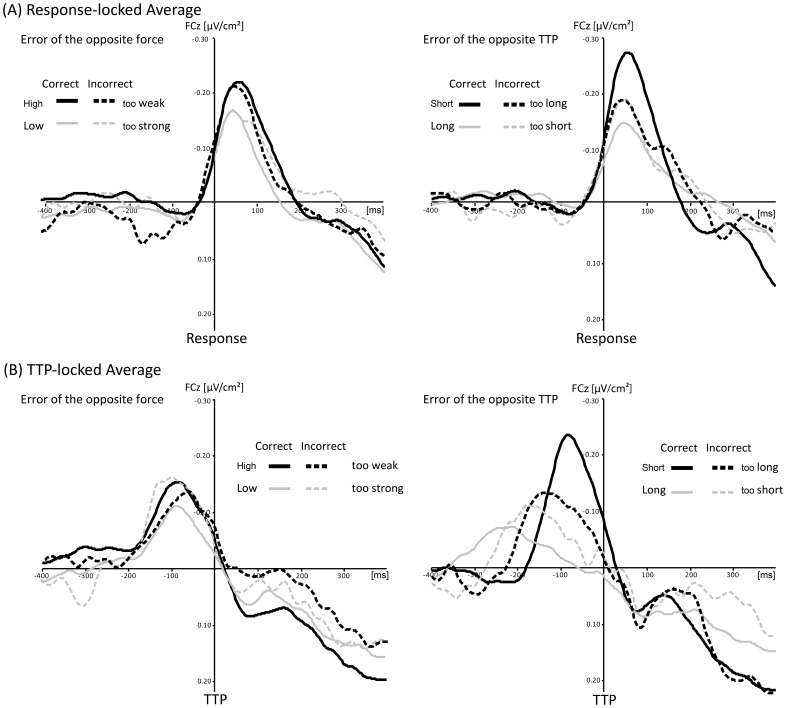
Grand average ERP waveforms for correct responses and errors of force selection. Grand average waveforms of event-related potentials at the FCz electrode site, (A) time-locked to response onset for (left panel) correct responses and errors of the opposite force and (right panel) for correct responses and errors of the opposite TTP, (B) time-locked to TTP onset for (left panel) correct responses and errors of the opposite force and (right panel) for correct responses and errors of the opposite TTP.

Figure 4A shows the mean CSD-ERP waveforms as a function of Target Force and Response Type. The *errors of the opposite force* (too strong for the low target force and too weak for the high target force, Figure 4A left panel) were averaged across the two TTP conditions as the number of error trials was too small for separate analyses. Analogously, the correct-response trials were also averaged across TTP conditions. In order to exclude a confounding effect of an unequal number of error trials, *chi^2^* tests were performed. These analyses showed no significant differences between the long and the short TTP condition for correct or incorrect responses (for all comparisons, *chi* ^2^ <1.0).

The two-way ANOVA for the mean MFN amplitudes yielded a significant main effect of Target Force, *F*(1, 21) = 14.2, *p*<.001. The post-hoc analyses showed that the MFN amplitude was significantly smaller in the low target-force condition (0.118±0.011 µV/cm^2^; *p*<.05) than in the high target-force condition (0.151±0.012 µV/cm^2^). There was no further significant difference in the MFN amplitude.

We further investigated *errors of the opposite TTP* (too short in the long TTP condition and too long in the short TTP condition, Figure 4 A right panel) and compared these errors types with correct responses. The conditions were averaged across the two force conditions. The number of trials did not differ significantly for the high and the low target-force condition (for all comparisons, *chi* ^2^ <1.0).

The two-way ANOVA showed a significant main effect of Target TTP, *F*(1, 21) = 23.4, *p*<.001, with smaller MFN amplitudes for the long (0.081±0.009 µV/cm^2^) than for the short TTP condition (0.107±0.010 µV/cm^2^). The interaction of Response Type and Target TTP was also significant, *F*(1, 21) = 4.2, *p*<.05. The difference between the long (0.061±0.011 µV/cm^2^) and the short TTP condition (0.102±0.017 µV/cm^2^; *p*<.05) was only significant for correct trials. There was no further significant difference (too short in the long condition: 0.110±0.016 µV/cm^2^; too long in the short condition: 0.105±0.021 µV/cm^2^).

### Time-to-peak Locked Average

The CSD-ERP waveforms were also averaged with respect to the TTP to investigate the MFN previous to the force peak (Figure 4B, left panel). Due to the TTP-locked averaging procedure and the high variability in the shape characteristics of the waveforms, we performed a peak-to-peak analysis to determine the MFN amplitude in the following. The two-way ANOVA including the *errors of the opposite force* revealed a significant main effect of Response Type, *F*(1, 21) = 23.7, *p*<.001, showing a higher MFN amplitude for incorrect (0.287±0.025 µV/cm^2^) than for correct (0.159±0.017 µV/cm^2^) responses. The significant interaction of the two factors, *F*(1, 21) = 10.9, *p*<.01, showed that the error “too strong” (0.330±0.034 µV/cm^2^) was significantly higher than the correct weak response (0.135±0.015 µV/cm^2^; *p*>.001). However, the difference in the high target-force condition was reversed (correct: 0.243±0.016 µV/cm^2^; too weak: 0.118±0.021 µV/cm^2^; *p*<.05).

The two-way ANOVA including the *errors of the opposite TTP* (Figure 4B, right panel) showed a significant effect of Response Type, *F*(1, 21) = 12.1, *p*<.01, with a higher MFN amplitude after incorrect (0.307±0.032 µV/cm^2^) than after correct responses (0.215±0.027 µV/cm^2^). The target TTP had also a significant effect on the MFN amplitude, *F*(1, 21) = 21.6, *p*<.001, with a higher MFN amplitude after short (0.303±0.031 µV/cm^2^) than after long TTP (0.218±0.024 µV/cm^2^). Finally, there was also a significant interaction between Response Type and Target TTP, *F*(1, 21) = 5.2, *p*<.05. The post-hoc analyses showed that the correct responses in the long TTP condition (0.132±0.017 µV/cm^2^) showed the smallest MFN amplitude compared to the other three conditions (incorrect long: 0.297±0.034 µV/cm^2^; correct short: 0.304±0.042 µV/cm^2^; incorrect short: 0.309±0.039 µV/cm^2^, for all comparisons, *p*s <.01).

## Discussion

In the present study, we investigated the relationship between two response parameters in a force-production task (i.e., PF and TTP) and the activity of the action-monitoring system reflected by the MFN. Previous studies reported inconsistent findings on whether this system monitors the accuracy of force productions. Whereas one study [3] reported increased monitoring activity after forceful responses (also for erroneous responses) compared to weak responses, our previous study [2] could not support these findings. As the two studies controlled the maximum force of the responses (PF) but not the time to reach this maximum (TTP), we suggested that different force-production mechanisms might have been involved in the different tasks. According to the PFUM [13], a higher response force can be produced by prolonging the duration of the force unit activation (i.e., an increased TTP of a force pulse) or by increasing the number of activated force units (i.e., no effect on TTP). Based on the comparison of the TTP variation in the two studies [2], [3], we hypothesized that the number and the duration of force units might affect the MFN amplitude and the MFN latency differentially. To test this hypothesis, we varied the two response parameters separately.

### Effects of Force Magnitude on the MFN

In contrast to our previous study [2] but in line with de Bruijn et al. [3], we found a clear difference in MFN amplitude between the high and the low target-force condition. The performance rates (ranging from 57% to 70%) demonstrate that the participants were capable of producing the target forces and the target TTPs. Differences between the present study and our previous study might be a result of performance differences, resulting from the employed precuing paradigm (i.e., precuing valid and invalid target-force information; PCRs: ±50%). The present results indicate that the participants were able to establish a representation of the correct degree of the required force, which is necessary for successful internal error detection [26], [27]. However, no difference between correct and incorrect responses in MFN amplitude was revealed in the first analysis of the present data after averaging across the different error types. Due to the present paradigm, we could distinguish twelve different error-type conditions, but the low number of trials in some conditions made it impossible to analyze them separately. However, as the errors of the opposite force (according to de Bruijn et al. error of selection) but not of the not-opposite force (according to de Bruijn et al. error of execution) had an increasing effect on the MFN amplitude [3], we performed a second set of analyses by contrasting correct responses with errors of the opposite force (as genuine errors of force selection could not occur in the present blocked design), which was the most frequent error type. However, there was no error-specific difference on the MFN amplitude in the response locked data. Only in the TTP-locked analysis, a difference between correct and incorrect response trials was revealed. Only in the low target-force condition, a significant higher MFN amplitude for errors of the opposite force (i.e., too strong responses) compared to correct weak responses was revealed. In the high target-force condition, converse findings were obtained, meaning that a slightly smaller MFN amplitude was shown for errors of the opposite force (i.e., too weak responses) compared to correct strong responses. Interestingly, de Bruijn et al. [3] also showed an increase of MFN for too strong responses, however, the MFN after too weak responses was not increased but also slightly, but not significantly decreased (see their Figure 3). The present results indicate that the degree of force is the crucial variable for the degree of MFN amplitude in a force-production task and that the degree of force can also account for the seemingly error-specific effect. The different findings of the previous studies might be due to controlling for TTP, as TTP turned out to be an MFN affecting variable (for more discussion, see below).

Based on the scalp distribution and the temporal occurrence of the MFN component, we assume that the component reflects the same or at least a similar processing system as Ne/ERN and CRN. In accordance with several studies [28], [29], [9], we could demonstrate that the MFN might represent more than the activity of error processing. It is important to note that our data did not disprove the possibility of successful force-error detection in specific force-production tasks (e.g., producing a tone of a specific loudness with a piano key by a practiced piano player), but most likely much more practice than two one-hour sessions might be required to learn the specific threshold of a correct, forceful response.

### Effects of TTP on the MFN

The relationship between the temporal dynamics of force productions and the MFN amplitude was examined by a systematic variation of TTP. As high response forces, which lead to an increase of MFN amplitude [3], are usually accompanied by long TTPs (e.g., [30]), we assumed that the monitoring system might be sensitive to temporal adjustments of force productions, and not (only) to the force magnitude per se. According to the assumptions of the PFUM [13], a slow rising time of a force pulse (i.e., long target-TTP condition, here: 300 to 500 ms after response onset) is accomplished by the prolongation of force-unit activation. Thus, we assumed that the monitoring activity increases because it is required for a longer period and we hypothesized that there would be a higher MFN amplitude after responses with a long TTP than after those with a short TTP (here, 113 to 188 ms after response onset), irrespective of the target-force condition. Regardless of the averaging procedure (response locked or TTP locked; Figures 3, and 4), an MFN appeared in all conditions. In contrast to our predictions, however, the present data clearly demonstrate that the MFN amplitude after responses with a long TTP was smaller than the MFN amplitude after responses with a short TTP. These findings indicated that the ACC is activated for a longer period in long TTP trials (see also longer MFN latencies) but with a smaller maximum.

The second analysis contrasting correct responses with errors of the opposite TTP showed a higher MFN amplitude in the too-short TTP condition compared to the correct long TTP condition but a smaller MFN amplitude for the too-long TTP condition compared to the correct short TTP condition. Thus, the monitoring system seems to be highly sensitive to the time required to reach the force magnitude (i.e., the longer the TTP the smaller the MFN) but not to the erroneous timing. Similar to the detection of the use of incorrect force parameters, the error detection of timing parameters of a response is possible but seems to be rather difficult, too. This was demonstrated by an RT-error detection task [31], in which the response-locked averaged MFN was only increased when a given response was extremely beyond a defined RT deadline (fourth quartile of the RT error distribution, i.e., >250 ms). The deviation of the incorrect TTP was much smaller (approximately ±50 ms) in the present study.

The presented relationship between the MFN amplitude and the magnitude of the response force, as well as the duration required to reach the maximum force, indicates increased monitoring activity with increasing force and reduced monitoring activity with prolonged force productions. Three alternative theoretical considerations are presented in the following.

### Ballistic vs. Guided Response Processing

One could assume that the ACC monitors ballistic response processes (required to produce force pulses with a short TTP) but not guided response processes (required to produce force pulses with a long TTP; see also [2]). Ballistic processes are defined as motor processes comprising a sequence of motor commands, which are assumed to be inevitable after their initiation. Guided processes, in contrast, are defined as controlled motor processes, which often use peripheral feedback to allow the adjustment of movements (e.g., [14]). Some authors assumed that the MFN-related monitoring process did not rely on peripheral feedback, as the MFN peaks too early to be influenced by peripheral feedback processes (see [32], [33]). They argued that it takes about 100 ms for a visual or a proprioceptive feedback to affect an ongoing movement [34]. Another study [35] showed that the error-related MFN can be elicited without peripheral feedback, because the MFN was also present after response errors in a deafferented patient. However, the authors [35] also argued that the time taken to *affect the cortex* should be considered and not the time taken to *affect the movement*. As cortical activation can be measured 20 ms after nerve stimulation in terms of somatosensory ERPs in primary motor and sensory areas [36], which also show connections with the ACC (for reviews, see [37], [38]), peripheral information is able to have fast access to the ACC but it is not necessary to evoke MFN. Thus, two different kinds of monitoring loops might be involved in monitoring short and long force pulses. If the correct production of a force pulse with long TTP requires peripheral feedback, one can assume that the additional feedback loops (in a range of 300 to 500 ms one can assume several loops) need more time than monitoring of a ballistic response, where no peripheral feedback is required. This might reduce the monitoring activity but not totally inhibit monitoring in long TTP trials.

In typical tasks investigating error processing (like a flanker task, Stroop task or other types of choice-reaction tasks) usually ballistic rather than controlled responses are required. Thus, a strong variation of response force and/or TTP is usually not given. Nevertheless, the response force or the TTP in a choice-reaction task might vary even without an explicit instruction due to differential experimental parameters. For example, it was shown that parameters like response conflict [9], stimulus probability [39], or the uncertainty of accuracy [40], [41] affect the MFN amplitude. Research on response force showed that the temporal uncertainty of stimulus presentation or variations in response preparation led to stronger responses [42], [43]. Furthermore, stimulus duration and stimulus intensity [44], and also response conflict [45], are related to the variations in the degree of response force. The MFN after response errors (i.e., Ne/ERN) is doubtless related to error processing in the above-mentioned tasks; however, some of the observed (unexplained) variations in Ne/ERN and CRN might be related to different variations in physical effort during response execution.

### Effects of Error Salience

Differences in the subjective significance of the responses could also explain the non-error-specific difference in the MFN amplitude (e.g., [4], [46]), indicating that the medial-frontal cortex is also involved in the evaluation of behavior (for an overview, see [47]). This consideration is supported by the structure of the ACC, which also shows - besides multifunctional connections involving sensory afferents and pathways to motor areas - connections to the limbic system (for reviews, see [48], [49]). Effects of the significance of response errors on the MFN were investigated by the use of a four-choice flanker task to employ different significant responses errors [50]. If the response error followed a flanker stimulus, it should be more important to the participants compared to a response error committed after a non-flanker stimulus, because it was violating the task’s goal of ignoring the flanker, which could be supported by their findings.

An effect of the significance or purposiveness of force magnitude on the activity of the motor cortex (lateralized readiness potential) was investigated by the following task [51]. After the participants were instructed to pull a trigger at an easy, unspecified force level (non-purposive response), they had to pull the trigger with an exact target force (purposive task), which was defined by the mean response force from the prior non-purposive task. The authors revealed that a specific response force led to a higher neuronal activity in the motor cortex in terms of motor-related potentials if it was purposive or significant to the participants. This is an interesting result, as high response forces were previously found to result in a higher activation of the motor cortex than low response forces (e.g., [52], [53]; for a converse finding, see [18]).

The participants in the present study might also have subjectively interpreted the responses in the high target-force condition as being more important, which would have resulted in the higher MFN amplitude after forceful responses compared to weak ones. As the findings on this effect are ambiguous [2]–[5], the influence of the functional significance of force magnitudes might be an explanation, and should be investigated more specifically.

### Force Unit Monitoring Model

**Figure 5:**
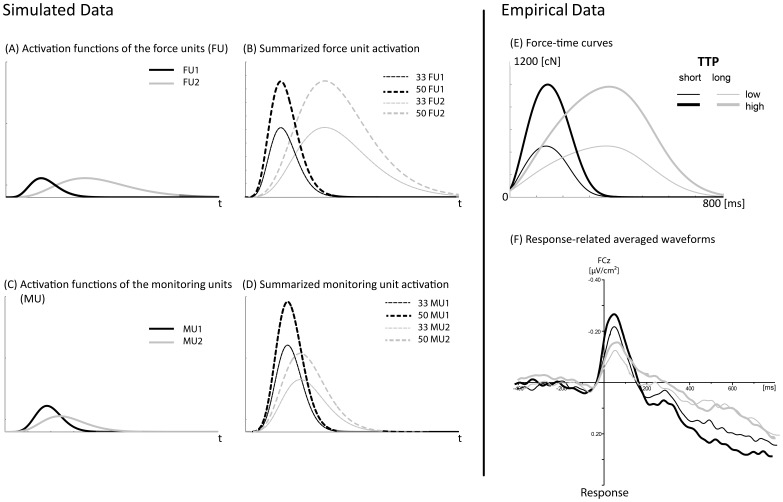
The force-unit monitoring model. Simulated data. (A) Two gamma functions with the same peak amplitude representing the force-unit activation for the short TTP (FU1) and the long TTP (FU2) condition. (B) The resulting function from the summation of the two 33 and 50 FU functions, representing the low and high target-force conditions and the short and long target-TTP conditions. (C) Two gamma functions with the same area under the curve representing the monitoring unit activation for the short TTP (MU1) and the long TTP (MU2) condition. (D) The resulting functions from the summation of the two 33 and 50 MU functions. Empirical data. (E) Mean force-time curves, time-locked to the response onset (0 ms) as a function of target force and target TTP. (F) Grand average waveforms of event-related potentials as a function of target TTP and target force.

The variations in shape and dynamics of the MFN component (slopes, area) across the conditions were also an interesting finding. The analysis of the slopes of the components’ rises and decays supported that the long TTP condition is accompanied by a significant slower increase and decay (see also Figure 3A), indicating a smaller maximal but prolonged monitoring activity. The MFN latencies additionally supported this consideration by longer peak latencies for the long TTP condition compared to the short TTP condition, with the longest peak latency for the correct high target force, long TTP condition. Based on the theoretical framework of the PFUM [13], the present data suggest that the ACC activity might also be differentially sensitive to the *number* and the *duration* of the activated force controlling motor units but different from our original expectations. In the following, we present a simple, descriptive mathematical model to account for the present findings (Figure 5).

Two hypothetical activation functions of two force units (FU1 and FU2) with the same magnitude but different durations are illustrated in Figure 5A. Following the PFUM [13], we postulate that the activation of force units with a short duration (FU1) occurs for the short TTP condition and the activation of force units with a long duration (FU2) occurs for the long TTP condition. Based on the employed percentage of MVF for the two conditions, we assume that about 33.3% of all force units fire in the low force condition and about 50.0% do so in the high force condition. Thus, the resulting force pulse is based on the summation of the activated force units across time (*t*):
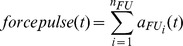
where *n_FU_* is the number of simultaneously activated force units (in the present numerical example, 33 or 50) and *a_FU_* is the activation of the force units (FU1 or FU2). A gamma function was used to simulate the activation function of the FU in our example, as gamma functions are mathematical functions that seem to be well-suited to representing physiological processes [54]. Figure 5B depicts the resulting four force pulse functions, which show a rather good (qualitative) fit with the empirical force data (Figure 5E).

It seems reasonable that more or fewer ACC neurons might be involved, depending on the task-specific *physical effort* expended on the monitoring system because more force producing motor units (FUs) and thus muscle fibers have to be monitored. The neural link between the dorsal ACC and the primary motor cortex as well as supplementary motor cortex [55] might be the crucial pathway between the FUs and the force-unit monitoring ACC units (here, MUs, i.e., a number of ACC neurons). In the following, we postulate that some monitoring units (MUs) within the ACC are more or less activated depending on the physical effort spent on the task. Based on the difference between the shapes of the force-time curves and the shapes of the MFN component, the MUs seem to follow a different activation pattern compared to the FUs. One could assume that the activation of a MU can differ in peak amplitude, peak latency, rise, and decay. The differences between the MU1 and MU2, in our descriptive example, are that MU1 (short monitoring) shows a steeper rise and a steeper decay and a higher peak amplitude compared to MU2 (long monitoring). Furthermore, assuming that the same monitoring activity is required in both units, but in different time periods, we postulated that the two areas under the curve (i.e., the integral of MU1 equals the integral of MU2) are equal (see Figure 5C). This would account for the reduction of the peak as well as slower rise and decay as shown for longer TTPs (correct and incorrect). Similar to the computation of force pulses, we assumed that the resulting monitoring activity is based on a summation of the activated MUs across time (*t*):
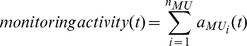
where *n_MU_* is the number of currently activated MUs (analogously, 33 or 50) and *a_MU_* is the activation of the MUs (MU1 or MU2). Figure 5D shows the resulting curves, which fit the characteristics of the MFN amplitude rather nicely in the different conditions (Figure 5F). Hence, the largest MFN peak amplitude was observed in the high-target force/short-TTP condition, followed by the low-target force/short-TTP condition, high-target force/long-TTP condition, and low-target force/long-TTP condition. Furthermore, the variation in the time of rise and in the time of decay across the four conditions is also in line with the empirical data. The slowest time of decay was observed for the high-target force/long-TTP condition. Thus, using the *force-unit monitoring model* (FUMM), we were able to describe the MFN activity in a similar way to how the production of force was described by PFUM, and to describe how a prolonged TTP led to a decreased MFN amplitude but not to generally impaired monitoring.

The FUMM is able to explain TTP- and response-force-related effects as well as the seemingly error-specific effects in the present study and the findings of de Bruijn’s research group [3]. The two studies found a higher MFN after too strong responses compared to correct weak responses (i.e. errors of the opposite force). Furthermore, a smaller MFN amplitude for too weak responses compared to correct strong responses was shown in the present study. According to the model, more MUs were activated in the error trials “too strong” and fewer MUs were activated in the error trials “too weak”.

An interesting question is whether the FUMM can explain the finding of weaker response force for hand errors than for correct responses [56], [57], which seems to contrast with the fact that usually there is a higher MFN after hand errors. It is important to note that a weaker response force for hand errors is discussed to be an indicator of an error correction/compensation mechanism [4]. The participant detects the hand error, tries to withhold the response during the execution, and hence responds less strongly. In that case, it is not the participant’s task to produce a specific amount of force, and force monitoring is less (or not) required. The error detection of a hand error is more salient than force monitoring, as was demonstrated by de Bruijn et al. [3]. Perhaps there is an overlap of the hand error related activity and the smaller force unit (monitoring) activity, which can be separated in future simulation studies.

Our new model can also contribute to the ongoing controversial discussion of whether Ne/ERN and CRN reflect general action monitoring activity, response-conflict monitoring or error-related information processing. In our understanding, Ne/ERN and CRN reflect the activity of general action monitoring, with error detection as one important part of successful action monitoring. Error detection can only be successful if clear and well distinguishable definitions/representations or “correct” and “incorrect” response parameters exist. In more challenging motor tasks, these representations have to be established across a longer period. The important contribution to this discussion is that a mathematical model, even this rather descriptive mathematical model (in its present form), provided unequivocal and thus testable assumptions. The FUMM can be tested by experimental variation as well as simulation studies. It can be combined with assumptions of other models of Ne/ERN like the response-conflict model [9] or the reinforcement-learning theory [58] by including additional parameters. Furthermore, to our knowledge the FUMM is the first model which provides predictions for the entire MFN activity and its dynamic, not only for the peak of the component. The FUMM model is also in line with recent findings that the ACC is sensitive to behavioral adaption in terms of continuous updating [59]. The FUMM and its general mathematical approach can be adapted to different tasks and experimental settings. As mentioned above, force is usually not a crucial variable in tasks investigating the MFN (Ne/ERN, CRN); nevertheless, the physical effort can vary across the tasks depending on different experimental parameters. Besides physical effort, in future approaches one could also model variations in cognitive effort, for example, like the variations in error salience or engagement in a trial [35], [60].

### Conclusions

The present study provides strong evidence for a relationship between force-production parameters (magnitude and TTP of an isometric force pulse) and the response-related MFN. These findings support the idea that the ACC activity, as reflected in the MFN component, in addition to its well-documented error-specific sensitivity, is also sensitive to variations in response dynamics. We presented a new mathematical model, the FUMM, which is able to explain the increasing effect of force magnitude on the MFN amplitude and the decreasing effect of TTP prolongation on the MFN amplitude by assuming slightly reduced but prolonged activation of monitoring units.

## References

[1] Falkenstein M, Hohnsbein J, Hoormann J (1991) Effects of crossmodal divided attention on late ERP components: II. Error processing in choice reaction tasks. Electroencephalography & Clinical Neurophysiology 78: 447–455. 1712280 10.1016/0013-4694(91)90062-9

[2] Armbrecht A-S, Gibbons H, Stahl J (2012) Monitoring force errors: Medial-frontal negativity in a unimanual force-production task. Psychophysiology 49: 56–72. 21895690 10.1111/j.1469-8986.2011.01282.x

[3] De Bruijn ERA, Hulstijn W, Meulenbroek RGJ, van Galen GP (2003) Action monitoring in motor control: ERPs following selection and execution errors in a force production task. Psychophysiology 40: 786–795. 14696732 10.1111/1469-8986.00079

[4] Gehring WJ, Goss B, Coles MGH, Meyer DE, Donchin E (1993) A neural system for error detection and compensation. Psychological Science 4: 385–390.

[5] Scheffers MK, Coles MGH, Bernstein P, Gehring WJ, Donchin E (1996) Event-related brain potentials and error-related processing: An analysis of incorrect responses to go and no-go stimuli. Psychophysiology 33: 42–53. 8570794 10.1111/j.1469-8986.1996.tb02107.x

[6] Carter CS, Braver TS, Barch DM, Botvinick MM, Noll D, et al (1998) Anterior cingulate cortex, error detection, and the online monitoring of performance. Science 280: 747–749. 9563953 10.1126/science.280.5364.747

[7] Ullsperger M, von Cramon DY (2001) Subprocesses of performance monitoring: A dissociation of error processing and response competition revealed by event-related fMRI and ERPs. NeuroImage 14: 1387–1401. 11707094 10.1006/nimg.2001.0935

[8] Dehaene S, Posner MI, Tucker DM (1994) Localization of a neural system for error detection and compensation. Psychological Science 5: 303–305.

[9] Botvinick MM, Braver TS, Barch DM, Carter CS, Cohen JD (2001) Conflict Monitoring and Cognitive Control. Psychological Review 108: 624–652. 11488380 10.1037/0033-295x.108.3.624

[10] Bartholow BD, Pearson MA, Dickter CL, Sher KJ, Fabiani M, et al (2005) Strategic control and medial frontal negativity: Beyond errors and response conflict. Psychophysiology 42: 33–42. 15720579 10.1111/j.1469-8986.2005.00258.x

[11] Roger C, Bénar CG, Vidal F, Hasbroucq T, Burle B (2010) Rostral cingulate zone and correct response monitoring: ICA and source localization evidences for the unicity of correct- and error-negativities. NeuroImage 51: 391–403. 20152906 10.1016/j.neuroimage.2010.02.005 PMC3939321

[12] Gehring WJ, Willoughby AR (2002) The medial frontal cortex and the rapid processing of monetary gains and losses. Science 295: 2279–2282. 11910116 10.1126/science.1066893

[13] Ulrich R, Wing AM (1991) A recruitment theory of force-time relations in the production of brief force pulses: The parallel force unit model. Psychological Review 98: 268–294. 2047514 10.1037/0033-295x.98.2.268

[14] Ulrich R, Wing AM, Rinkenauer G (1995) Amplitude and duration scaling of brief isometric force pulses. Journal of Experimental Psychology: Human Perception and Performance 21: 1457–1472. 11766938

[15] Newell KM, Carlton LG (1988) Force variability in isometric responses. Journal of Experimental Psychology: Human Perception and Performance 14: 37–44. 2964505

[16] Schmidt RA, Zelaznik H, Hawkins B, Frank JS, Quinn JT Jr (1979) Motor-output variability: A theory for the accuracy of rapid motor acts. Psychological Review 86: 415–451. 504536

[17] van Galen GP, de Jong WP (1995) Fitts’ law as the outcome of a dynamic noise filtering model of motor control. Human Movement Science 14: 539–571.

[18] Sommer W, Leuthold H, Ulrich R (1994) The lateralized readiness potential preceding brief isometric force pulses of different peak force and rate of force production. Psychophysiology 31: 503–512. 7972605 10.1111/j.1469-8986.1994.tb01054.x

[19] Rinkenauer G, Ulrich R, Wing AM (2001) Brief bimanual force pulses: Correlations between the hands in force and time. Journal of Experimental Psychology: Human Perception and Performance 27: 1485–1497. 11766938

[20] Jasper HH (1958) The ten-twenty electrode system of the International Federation. Electroencephalography and Clinical Neuropsychology 10: 370–375. 10590970

[21] Gratton G, Coles MGH, Donchin E (1983) A new method for off-line removal of ocular artifact. Electroencephalography and Clinical Neurophysiology 55: 468–484. 6187540 10.1016/0013-4694(83)90135-9

[22] Pernier J, Perrin F, Bertrand O (1988) Scalp current density fields: concepts and properties. Electroencephalogr Clin Neurophysiol 69: 385–389. 2450736 10.1016/0013-4694(88)90009-0

[23] Perrin F, Bertrand O, Pernier J (1987) Scalp current density mapping: value and estimation from potential data. IEEE Trans Biomed Eng 34: 283–288. 3504202 10.1109/tbme.1987.326089

[24] Perrin F, Pernier J, Bertrand O, Echallier JF (1989) Spherical splines for scalp potential and current density mapping. Electroencephalogr Clin Neurophysiol 72: 184–187. 2464490 10.1016/0013-4694(89)90180-6

[25] Geisser S, Greenhouse SW (1958) An extension of Box’s results on the use of the F distribution in multivariate analysis. The Annals of Mathematical Statistics 29: 885–891.

[26] Falkenstein M, Hoormann J, Christ S, Hohnsbein J (2000) ERP components on reaction errors and their functional significance: A tutorial. Biological Psychology 51: 87–107. 10686361 10.1016/s0301-0511(99)00031-9

[27] Rabbitt PM (1966) Errors and error correction in choice-response tasks. Journal of Experimental Psychology 71: 264–272. 5948188 10.1037/h0022853

[28] Vidal F, Hasbroucq T, Grapperon J, Bonnet M (2000) Is the ‘error negativity’ specific to errors? Biological Psychology 51: 109–128. 10686362 10.1016/s0301-0511(99)00032-0

[29] Vidal F, Burle B, Bonnet M, Grapperon J, Hasbroucq T (2003) Error negativity on correct trials: A reexamination of available data. Biological Psychology 64: 265–282. 14630407 10.1016/s0301-0511(03)00097-8

[30] Newell KM, Carlton LG (1985) On the relationship between peak force and peak force variability in isometric tasks. Journal of Motor Behavior 17: 230–241. 15140693 10.1080/00222895.1985.10735346

[31] Stahl J (2011) Error detection and the use of internal and external error indicators: An investigation of the first-indicator hypothesis. International Journal of Psychophysiology 77: 43–52. 10.1016/j.ijpsycho.2010.04.005 20417668

[32] Coles MGH, Scheffers MK, Holroyd CB (2001) Why is there an ERN/Ne on correct trials? Response representations, stimulus-related components, and the theory of error-processing. Biological Psychology 56: 173–189. 11399349 10.1016/s0301-0511(01)00076-x

[33] Swick D, Turken U (2002) Dissociation between conflict detection and error monitoring in the human anterior cingulate cortex. Proceedings of the National Academy of Sciences of the United States of America 99: 16354–16359. 12456882 10.1073/pnas.252521499 PMC138615

[34] Jeannerod M (1988) The Neural and Behavioural Organization of Goal-Directed Movements. Oxford: Oxford University Press.

[35] Allain S, Hasbroucq T, Burle B, Grapperon J, Vidal F (2004) Response monitoring without sensory feedback. Clinical Neurophysiology 115: 2014–2020. 15294203 10.1016/j.clinph.2004.04.013

[36] Bötzel K, Ecker C, Schulze S (1997) Topography and dipole analysis of reafferent electrical brain activity following the Bereitschaftspotential. Experimental Brain Research 114: 352–361. 9166924 10.1007/pl00005643

[37] Paus T (2001) Primate anterior cingulate cortex: Where motor control, drive and cognition interface. Nature Reviews Neuroscience 2: 417–424. 11389475 10.1038/35077500

[38] Vogt BA, Finch DM, Olson CR (1992) Functional heterogeneity in cingulate cortex: The anterior executive and posterior evaluative regions. Cerebral Cortex 2: 435–443. 1477524 10.1093/cercor/2.6.435-a

[39] Suzuki K, Shinoda H (2011) Probability effects of response and stimulus on error-related negativity. Neuroreport: For Rapid Communication of Neuroscience Research 22: 902–905. 10.1097/WNR.0b013e32834cd736 22027513

[40] Scheffers MK, Coles MGH (2000) Performance monitoring in a confusing world: Error-related brain activity, judgments of response accuracy, and types of errors. Journal of Experimental Psychology: Human Perception & Performance 26: 141–151. 10696610 10.1037//0096-1523.26.1.141

[41] Pailing P, Segalowitz S (2004) The effects of uncertainty in error monitoring on associated ERPs. Brain and Cognition 56: 215–233. 15518937 10.1016/j.bandc.2004.06.005

[42] Mattes S, Ulrich R (1997) Response force is sensitive to the temporal uncertainty of response stimuli. Perception & Psychophysics 59: 1089–1097. 9360481 10.3758/bf03205523

[43] Mattes S, Ulrich R, Miller J (1997) Effects of response probability on response force in simple RT. Quarterly Journal of Experimental Psychology: Section A 50: 405–420.

[44] Stahl J, Rammsayer TH (2005) Accessory stimulation in the time course of visuomotor information processing: Stimulus intensity effects on reaction time and response force. Acta Psychologica 120: 1–18. 16098832 10.1016/j.actpsy.2005.02.003

[45] Kantowitz BH (1973) Response force as an indicant of conflict in double stimulation. Journal of Experimental Psychology 100: 302–309. 4745460 10.1037/h0035780

[46] Hajcak G, Holroyd CB, Moser JS, Simons RF (2005) Brain potentials associated with expected and unexpected good and bad outcomes. Psychophysiology 42: 161–170. 15787853 10.1111/j.1469-8986.2005.00278.x

[47] Ridderinkhof KR, Ullsperger M, Crone EA, Nieuwenhuis S (2004) The role of the medial frontal cortex in cognitive control. Science 306: 443–447. 15486290 10.1126/science.1100301

[48] Devinsky O, Morrell MJ, Vogt BA (1995) Contributions of anterior cingulate cortex to behaviour. Brain: A Journal of Neurology 118: 279–306. 7895011 10.1093/brain/118.1.279

[49] Öngür D, Price JL (2000) The organization of networks within the orbital and medial prefrontal cortex of rats, monkeys and humans. Cerebral Cortex 10: 206–219. 10731217 10.1093/cercor/10.3.206

[50] Maier M, Steinhauser M, Hübner R (2008) Is the error-related negativity amplitude related to error detectability? Evidence from effects of different error types. Journal of Cognitive Neuroscience 20: 2263–2273. 18457501 10.1162/jocn.2008.20159

[51] Masaki H, Takasawa N, Yamazaki K (1998) Enhanced negative slope of the readiness potential preceding a target force production task. Electroencephalog Clin Neurophysiol 4: 390–397. 10.1016/s0168-5597(98)00019-7 9714381

[52] Kristeva R, Kornhuber HH (1980) Cerebral potentials related to the smallest human finger movement. Progress in Brain Research 54: 177–182. 7220913

[53] Slobounov SM, Ray WJ, Simon RF (1998) Movement-related potentials accompanying unilateral finger movements with special reference to rate of force development. Psychophysiology 35: 537–548. 9715098 10.1017/s0048577298970342

[54] Stahl J, Gibbons H, Miller J (2010) Modeling single-trial LRP waveforms using gamma functions. Psychophysiology 47: 43–56. 19674390 10.1111/j.1469-8986.2009.00878.x

[55] Galléa C, de Graaf J, Pailhous J, Bonnard M (2008) Error processing during online motor control depends on the response accuracy. Behavioural Brain Research 193: 117–125. 18584891 10.1016/j.bbr.2008.05.014

[56] Rabbitt, P M (1978) Detection of errors by skilled typists. Ergonomics 21(11): 945–958.

[57] Allain S, Carbonnell L, Burle B, Hasbroucq T, Vidal F. On-line executive control: An electromyographic study. Psychophysiology 41(1): 113–116. 14693006 10.1111/j.1469-8986.2003.00136.x

[58] Holroyd CB, Coles MGH (2002) The neural basis of human error processing: Reinforcement learning, dopamine, and the error-related negativity. Psychological Review 109: 679–709. 12374324 10.1037/0033-295X.109.4.679

[59] Sheth SA, Mian MK, Patel SP, Asaad WF, Williams ZM, Dougherty DD, Bush G & Eskandar EN (2012) Human dorsal anterior cingulate cortex neurons mediate ongoing behavioural adaptation. Nature 488: 218–22. 22722841 10.1038/nature11239 PMC3416924

[60] Cavanagh J, Frank M, Klein T, Allen J (2009) Frontal theta links prediction errors to behavioral adaptation in reinforcement learning. NeuroImage 49: 3198–3209. 19969093 10.1016/j.neuroimage.2009.11.080 PMC2818688

